# Low coverage but few inclusion errors in Burkina Faso: a community-based targeting approach to exempt the indigent from user fees

**DOI:** 10.1186/1471-2458-10-631

**Published:** 2010-10-21

**Authors:** Valéry Ridde, Slim Haddad, Béatrice Nikiema, Moctar Ouedraogo, Yamba Kafando, Abel Bicaba

**Affiliations:** 1 Research Centre of the University of Montreal Hospital Centre (CRCHUM), Canada; 2 Department of Social and Preventive Medicine, University of Montreal, Canada; 3 Institut de recherche en sciences de la santé (IRSS) du CNRST, Burkina Faso; 4 Agence de formation, de recherche et d'expertise en santé pour l'Afrique (AFRICSANTE), Burkina Faso; 5 Société d'étude et de recherche en santé publique (SERSAP), Burkina Faso

## Abstract

**Background:**

User fees were generalized in Burkina Faso in the 1990 s. At the time of their implementation, it was envisioned that measures would be instituted to exempt the poor from paying these fees. However, in practice, the identification of indigents is ineffective, and so they do not have access to care. Thus, a community-based process for selecting indigents for user fees exemption was tested in a district. In each of the 124 villages in the catchment areas of ten health centres, village committees proposed lists of indigents that were then validated by the health centres' management committees. The objective of this study is to evaluate the effectiveness of this community-based selection.

**Methods:**

An indigent-selection process is judged effective if it minimizes inclusion biases and exclusion biases. The study compares the levels of poverty and of vulnerability of indigents selected by the management committees (n = 184) with: 1) indigents selected in the villages but not retained by these committees (n = 48); ii) indigents selected by the health centre nurses (n = 82); and iii) a sample of the rural population (n = 5,900).

**Results:**

The households in which the three groups of indigents lived appeared to be more vulnerable and poorer than the reference rural households. Indigents selected by the management committees and the nurses were very comparable in terms of levels of vulnerability, but the former were more vulnerable socially. The majority of indigents proposed by the village committees who lived in extremely poor households were retained by the management committees. Only 0.36% of the population living below the poverty threshold and less than 1% of the extremely poor population were selected.

**Conclusions:**

The community-based process minimized inclusion biases, as the people selected were poorer and more vulnerable than the rest of the population. However, there were significant exclusion biases; the selection was very restrictive because the exemption had to be endogenously funded.

## Background

Requiring payment for healthcare services limits access to care for the worst-off [1,2]. Given that countries are not about to stop charging for services, and in the absence of social insurance, some countries propose to exempt the worst-off from user fees [3-5]. The first targets of such exemption measures are the indigent, i.e., those with a "*sustained incapacity to pay for minimum health care" *[6]. While this idea may seem simple enough, its implementation is confronted with enormous challenges in practice [6-8]. A key challenge is the selection of indigents, about whom very little is known in Africa, since there have been few studies [8]. The ideal selection process has to be feasible, reproducible, inexpensive, and well understood and accepted by those implementing it [8-11]. It also needs to be effective, i.e., it must minimize inclusion and exclusion biases in order to guarantee optimal use of public resources [12]. Studies to assess the effectiveness of exemption mechanisms that were tried are still rare and their results are inconsistent [8,9]. Thus, decision-makers have no yardstick, nor any sufficient factual bases to support them in choosing an optimal method for indigent selection.

Burkina Faso is ranked sixth-to-last in the 2009 Human Development Index. In 1993, the country launched the Bamako Initiative, which generalized the charging of user fees for services provided at public healthcare facilities. Substantial investments have been made since 1993 to support the reforms and improve the quality of and access to services [13]. In 2008, 8.4% of the government's budget was allocated to the Ministry of Health. Public healthcare is organized in a classical pyramidal model. First-contact services consist of primary health centres (called CSPSs) that include a dispensary, a maternity centre and an essential medications depot. Each CSPS is overseen by a management committee (COGES) whose members are elected by the community. In particular, these committees are responsible for managing the funds collected through cost recovery schemes, since patients must pay for medications and consultations. Medical Centres with a surgical ward (district hospitals) constitute the second level in any given district. The third level comprises nine regional hospitals and two national hospitals.

The government of Burkina Faso decided to exempt indigents from paying for services in primary care health centres when it generalized user fees in the 1990 s [14]. Government credits were allocated but, as in other countries of the region [15], this strategy was slowed down by a lack of consensus on implementation mechanisms and a technocratic process which until then had been rather ineffective [16-18].

This is why a community-based indigent targeting experience was undertaken in 2007 in the rural district of Ourgaye (260,000 inhabitants in 2006) [19]. The process included the implementation of 124 village selection committees (VSC) located in the catchment areas of 10 CSPSs. VSCs were asked to agree on a list of persons to be considered indigents. In a prior workshop involving all stakeholders (including community members), consensus was reached on a definition of the indigents who were to be selected in the villages: "*someone who is extremely disadvantaged socially and economically, unable to look after himself (herself) and devoid of internal or external resources*." A first round led to the selection of 566 indigents by the VSCs. The names of the indigents were then communicated to the members of the 10 COGESs, who retained about half (n = 269) of them. For this selection, the COGESs had no more specific criteria than the statement above, nor did they propose any new criteria. Their choices were thus based on the same definition of indigence as that used by the VSCs. Indigents were provided with exemption cards with the approval of the provincial branch of the Department of Social Affairs, card holders being eligible for free care in public facilities. The implementation analysis demonstrated the feasibility and acceptability of the community-based approach promoted. The mechanism was set up quite rapidly (the whole process took about four months), at modest cost, and was well received by local stakeholders [19]. This article presents the evaluation of the effectiveness of this community-based approach.

## Methods

### Design

The evaluation of effectiveness is based on an analysis of how successful the selection processes are at: i) including households that are poor or extremely poor; and ii) not including those that are not poor or not extremely poor. In other words, a selection of indigents is considered effective if it minimizes inclusion biases (selection of non-indigents) and exclusion biases (not selecting indigents).

Our study compares the indigents selected by the COGESs (referred to hereafter as COGES indigents) using the community-based approach described above with three population groups. The first group consists of the indigents who were initially selected by the VSCs but did not make it into the COGESs' final selection (hereafter called non-retained indigents). The second group is a sample of service users who were designated as indigent by the nurses in charge of health centres (called ICP) in order to receive free drugs provided by the Ministry of Health, without any specific criteria having been applied (hereafter called ICP indigents). The third group is made up of rural households that participated in a national survey on living conditions (hereafter referred to as Quibb and rural households) that was conducted to develop a socio-economic profile of the country's households [20].

### Hypotheses

Comparing these four groups to evaluate the effectiveness of the indigent selection process allows us to posit four research hypotheses (H). These hypotheses are based on communities' presumed effectiveness at identifying indigents as compared with the health workers, who do not come from these communities and therefore, *a priori*, have a less precise understanding of the levels of poverty. Moreover, given that the COGESs have the role of validating the village selection, the hypothesis is that they carry out this role effectively.

H1 = the COGES indigents are poorer and more vulnerable than those in the other three groups;

H2 = the COGES and non-retained indigents are poorer than the rural households (Quibb);

H3 = the COGES indigents and the non-retained indigents are poorer and more vulnerable than the ICP indigents;

H4 = targeting favours the worst-off.

### Selection of participants

There were 269 COGES indigents; 23 could not be found, 39 were children, two had died before the survey, and 20 were deaf and/or dumb and could not be interviewed. In the end, 184 COGES indigents were interviewed. Because of cost constraints, research on the non-retained indigents could only be carried out in three of the 10 CSPSs covered by the community-based selection process; 63 of the 297 non-retained indigents lived there, of which two were children and 48 could be met. We were able to interview 82 ICP indigents identified from the CSPS consultation registers; the reference population for this latter group is unknown because the ICPs did not systematically record the users' names and addresses.

### Questionnaires, outcome indicators

To develop the socio-economic profiles of the households of the three indigent groups, we used the same questionnaire as was used for the national standard of living surveys (Quibb [20]). Following the National Institute of Statistics standards, household income was estimated on the basis of the household annual consumption per capita. This first questionnaire also included indicators of household size and structure, means of production, and characteristics of the house and living environment. A second questionnaire was also used only for the indigents to determine their sociodemographic characteristics, physical and mental capacities, and ability to satisfy basic food and health-related needs. To calculate the standard of living of rural households (Quibb) at the time of the survey (2007), we used the database of the 2003 survey, with the income measures (still estimated based on consumption) annualized on the basis of inflation, because later surveys did not provide such measures.

### Analyses

Comparisons of the socio-economic profiles of households in which the indigents resided and of certain basic needs such as income, access to healthcare or to food (measure of vulnerability) in the three groups of indigents were based on chi-square testing (partial or complete tests) or mean comparison testing (ANOVA). Economic poverty in each group is determined by analysis of income distribution in the groups and mean testing comparing the incidence of poverty (proportion of poor in the group or Head Count Index) and the depth of the poverty (mean poverty of the poor measured by the average gap between the income of the poor and the poverty threshold).

The effectiveness of the targeting was assessed with respect to how well the selection process was able to minimize inclusion and exclusion biases. This comparative approach requires using reference measures for judging people's eligibility for exemption from payment. We used two criteria to assess potential eligibility for exemption: the poverty line and the extreme poverty line. In Burkina Faso, poverty lines are calculated based on the cost-of-basic-needs approach [21]. The poverty line takes into account both the food and non-food needs of households and enables the identification of poor households (L = 82,672 F CFA in 2003, or 170 US$ at that time). The national incidence of poverty in 2003 was 46%. The food poverty line corresponds to the value of the caloric intake required to satisfy average daily caloric needs (2,283 calories per person per day). This line is used as the threshold value to identify extremely poor households (L = 41,153 F CFA in 2003, or $85 US at that time), for an incidence of extreme poverty in 2003 of 9% [22]. For our analyses, poverty lines were adjusted to take into account the Consumer Price Indexes (CPI) from 2004 to 2007.

The rate of coverage corresponds to the number of beneficiaries who were selected and met the criteria (poverty and extreme poverty) in relation to the total number of inhabitants in the villages involved who met the criteria. The targeting coefficient measures the gap between the indigent and non-indigent coverages and provides an indication of targeting efficacy. Targeting is considered perfect if the coefficient equals 1. It is progressive (in favour of the poor) when the value of the coefficient is positive and regressive when the coefficient is negative.

## Results

### Inclusion of non-poor persons and the profile of households from which indigents come

**Figure 1:**
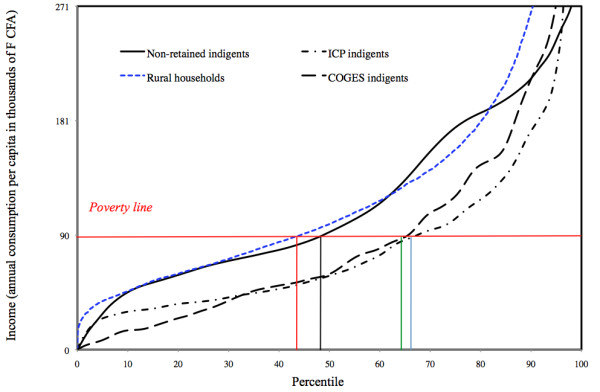
**Comparison of poverty among ICP indigents, COGES indigents, non-retained indigents and rural households**. Figure 1 show the income distributions in three groups of indigents and the Quibb households.

Households in which the three indigent groups lived appeared to be more vulnerable and poorer than the reference rural households (Table 1; contrasts (1,2,3) vs. (4)). The income distributions in these three groups were also different from that of the Quibb households (Figure 1). Thus, the COGESs, the VSCs and the ICPs all tended to minimize inclusion biases.

**Table 1:** Socio-economic characteristics of the households of indigents (ICP, COGES, non-retained) and of the rural population (Quibb)

**Indigent group**	**ICP**	**COGES**	**Non-retained**	**Quibb**	**Contrasts**^**&**^**(pValue)**		
	
**Household characteristics**	**(1)** **(n = 129)**	**(2)** **(n = 197)**	**(3)** **(n = 48)**	**(4)** **(n = 5900)**	**(1)** **vs. (2)**	**(1,2,3)** **vs. 4**	**(2)** **vs. (3)**
	
Mean number of household members	7.5 (6.8-8.2)	5.5 (4.8-6.2)	4.7 (3.9-5.5)	6.7 (6.6-6.8)	< 0.001	0,007	0,028
Female head of household	15.5%	27.4%	12.5%	6.3%	0.012	< 0.001	0.031
Health centre more than an hour away on foot	26.4% (18.7-34.1)	50.3% (43.2-57.3)	52.1% (37.4-66.7)	44.3% (43.0-45.6)	< 0.001	0.440	0.821
Possession of small ruminant animals	71.5% (63.7-79.4)	38.2% (31.4-45.0)	60.4% (46.1-74.8)	74.7% (73.6-75.8)	< 0.001	< 0.001	0.006
Possession of large livestock	41.5% (33.0-50.1)	20.1% (14.5-25.7)	29.2% (15.8-42.5)	62.0% (60.8-63.3)	< 0.001	< 0.001	0.186
Possession of a cart or plow	40.0% (31.5-48.5)	14.1% (9.2-18.9)	29.2% (15.8-42.5)	59.1% (57.8-60.3)	< 0.001	< 0.001	0.014
Possession of a radio	43.8% (35.2-52.5)	14.1% (9.2-18.9)	20.8% (8.9-32.8)	37.9% (36.7-39.2)	< 0.001	< 0.001	0.258
	
Mean annual consumption by head ‡	89,521	93,238	118,475	148,076	0.737	< 0.001	0.082
	(71,707-107,335)	(79,989-106,487)	(98,647-138,302)	(142,267-153,885)			
Median annual consumption by head ‡	59,423	59,043	96,951	98,969			
Proportion of food expenses	71.1%	61.8%	68.4%	57.0%	0.001	< 0.001	0.138
	(68.3-73.9)	(57.6-65.9)	(62.8-74.0)	(56.6-57.4)			
	
Incidence of poverty	66.7%	66.0%	47.9%	44.2%	0.899	< 0.001	0.020
	(58.4-74.9)	(59.3-72.7)	(33.3-62.6)	(43.0-45.5)			
Incidence of extreme poverty	35.7%	33.5%	6.3%	9.2%	0.688	< 0.001	< 0.001
	(27.3-44.0)	(26.9-40.2)	(0.0-13.4)	(8.5-10.0)			
Poverty deficit (CFA francs) ‡	43,039	47,386	28,372	29,017	0.165	< 0.001	0.001
	(39,127-46 952)	(43,071-51,700)	(20,551-36,193)	(28,338-29,696)			

Key: ‡1F CFA = 0. 00220 US$ at December 31, 2007; &: Partial Chi-Square or F-Test depending on the metrics of the outcome.

Table 1 compare the socio-economic characteristics of the households of indigents and of the rural population

### COGES indigents vs. ICP indigents

The GOGES indigents tended to live in households that had fewer possessions than did those of the ICP indigents (Table 1; contrasts (1) vs. (2)). However, the two groups were very comparable in the distributions of their respective incomes (Figure 1) and their levels of poverty (Table 1; contrasts (1) vs. (2)). We cannot therefore conclude *a priori *that the targeting done by the COGESs resulted in the selection of indigents from more economically disadvantaged households. On the other hand, a review of the personal characteristics of the indigents themselves showed significant differences (Table 2; contrasts (1) vs. (2)). The COGES indigents were more socially vulnerable: they were older, at greater risk of social isolation (widows and widowers), in worse health, and more often reported having experienced difficulty in accessing health facilities because of insufficient means.

**Table 2:** Comparison of indigents' personal socio-economic characteristics

Group	ICP	COGES	Non-retained	Contrasts (pValue)		
Personal characteristics	(1) (n = 82)	(2) (n = 184)	(3) (n = 48)	(1) vs. (2)	(2) vs. (3)	(1,2) vs. (3)
	
Average age	52.8 (48.8-56.7)	64.2 (61.8-66.5)	64.4 (59.6-69.3)	< 0.001	0.932	0.170
Proportion 60 years old +	35.4% (24.8-45.9)	69.0% (62.3-75.8)	70.8% (57.5-84.2)	< 0.001	0.809	0.113
Proportion of widowers - widows	34.6% (24.0-45.1)	46.7% (39.5-54.0)	39.6% (25.2-53.9)	0.066	0.377	0.659
Proportion of females	47.6% (36.5-58.6)	46.7% (39.5-54.0)	47.9% (33.3-62.6)	0.902	0.885	0.906
Not educated	93.8% (88.3-99.2)	96.2% (93.4-99.0)	95.8% (90.0-100)	0.395	0.908	0.912
	
Suffering from a disability	42.5% (31.4-53.6)	61.4% (54.3-68.5)	62.5% (48.3-76.7)	0.004	0.891	0.382
Health problem of more than 6 months	55.0% (43.9-66.1)	76.6% (70.5-82.8)	72.9% (59.9-86.0)	< 0.001	0.594	0.692
	
No income generating activity in the past 7 days	73.8 (63.9-83.6)	81.5 (75.9-87.2)	97.9 (93.7-100)	0.154	0.005	0.002
Difficulty in meeting daily food needs	32.5% (22.0-43.0)	35.9% (28.9-42.9)	41.7% (27.2-56.1)	0.599	0.461	0.367
	
Had to ask someone else for food in the past 30 days	26.3% (16.4-36.1)	36.4% (29.4-43.4)	20.8% (8.9-32.8)	0.108	0.041	0.087
Had to ask for help to pay for healthcare in the past 6 months	30.0% (19.7-40.3)	37.0% (29.9-44.0)	20.8% (8.9-32.8)	0.277	0.035	0.057
Unable to pay for drugs for a child	28.8 (18.6-38.9)	28.3 (21.7-34.8)	16.7 (5.7-27.6)	0.936	0.103	0.091
Sold animals or land to pay for drugs in the past 6 months	8.8 (2.4-15.1)	9.2 (5.0-13.5)	6.3 (0.0-13.4)	0.899	0.513	0.521
Delayed going to the dispensary because of lack of funds in the past 6 months	28.8 (18.6-38.9)	47.8 (40.5-55.1)	39.6 (25.2-53.9)	0.004	0.310	0.751

&: Partial Chi-Square or F-Test depending on the metrics of the outcome.

Table 2 compare the personal characteristics of the indigents

### COGES indigents vs. non-retained indigents

The COGESs retained only half of the people on the lists transmitted to them by the VSCs (269/566). The majority of indigents in both groups were over the age of 60, which would appear to reflect a particular sensitivity on the part of the committees to the precarious conditions in which the elderly lived. The economic criterion was clearly a determining factor in the selection. The retained indigents lived in families that were clearly poorer (Table 1). The majority of indigents proposed by the VSCs who lived in extremely poor households, however, were retained by the COGESs. Thus, barely 6% of the non-retained indigents lived in extremely poor households, which was a smaller proportion than that of the reference population. Table 2 confirms that the economic criterion was a determining factor in the triage conducted by the COGESs. The two groups show differences in only three of the 14 indicators that were used to compare them (Table 2; contrast (2) vs. (3)). These three indicators are more indicative of people's ability to pay than of their social condition or health needs: no income generating activity in the past 7 days; had to ask someone else for food in the past 30 days; had to ask for help to pay for healthcare in the past 6 months.

### Exclusion of the poor

Whatever the eligibility criteria, the low level of coverage of the poor is enlightening. Less than 1% of the extremely poor population was selected (Table 3). This proportion drops to 0.36% for the population living below the poverty line. The extreme moderation of the targeting coefficients, both near zero, clearly represents the very restrictive nature of the COGESs' selection processes.

**Table 3:** Coverage and effectiveness of indigent targeting according to eligibility criteria applied

	Coverage			Effectiveness of targeting	
				**Targeting coefficient**	

	**Eligible**	**Non-eligible**		**Ratio**	**Difference**

**Eligibility criteria**	**(1)**	**(2)**	**Total**	**(1)/(2)**	**(1)-(2)**
	
**Poverty line**					
Number of individuals	42,367	53,486	95,853		
Number of beneficiaries	153	92	245		
		
**Coverage (A)**	**0.36%**	**0.17%**	**-**	**2.1**	**0.19%**
	
**Extreme poverty line**					
Number of individuals	8,818	87,035	95,853		
Number of beneficiaries	69	176	245		
		
**Coverage (B)**	**0.78%**	**0.20%**		**3.9**	**0.58%**

**Coverage ratio (B)/(A)**	**2.17**	**1.18**	**-**	**1.84**	**3.07**

Table 3 show the coverage ratios between eligible and non-eligible populations

That being said, looking at the coverage ratios between eligible and non-eligible populations confirms what was predicted by the comparisons between groups. While the targeting is very restrictive, it is also effective in terms of minimizing inclusion biases (Table 3). Coverage of the poor is twice that of the non-poor (0.36% vs. 0.17%), and coverage of the extremely poor is four times greater than that of the rest of the population (0.78% vs. 0.20%).

## Discussion

### Methodological limitations

We identified the poor by comparing their levels of consumption against the reference measure, which was the poverty line as established by the government authorities. This approach tends to look at poverty essentially from an economic standpoint in a rural context where the concept of poverty is relative and complex to comprehend [23]. This community-based experiment was conducted exclusively in a rural setting, and therefore the results must be considered in this particular context. We cannot say anything about the feasibility and effects of this type of community-based experiment in an urban setting, which remains to be tested. A second limitation is related to our small sample sizes, which do not allow for detailed analyses such as stratified analyses in population subgroups. Fortunately, the different indicators provide convergent results that strengthen the internal validity of the study.

### A community-based process that minimizes inclusion biases

In a previous article [19], we described how this participative process for selecting indigents was considered appropriate and was much appreciated by the social actors. The present study shows that this community-based process is, additionally, potentially effective with respect to its first criterion which is the ability to minimize inclusion biases in the identification of people who should benefit from the exemption. In fact, the results suggest that the COGESs appropriately controlled inclusion biases when they prepared their final list from the names of indigents proposed by the VSCs.

They were able to retain people from households living in extreme poverty and from very significantly vulnerable situations. This bodes well, since many studies have demonstrated the ineffectiveness of administrative processes for selecting indigents in Africa [18] and in Burkina Faso [16,17]. Equally numerous are the authors who promote such community-based approaches [8,10] without necessarily having sufficient evidence. In another context, in Cambodia, three experiences were also found to be effective in reducing inclusion errors. One of these demonstrated that a community-based process was definitely more effective in selecting the extremely poor [24], which we also observed to some extent in this study.

In this rural experience, it would also be particularly useful to understand better, by means of qualitative studies, the process that led to the selection of a majority of elderly persons. In a social context where intergenerational solidarity is disintegrating [25], the care of the elderly by means of social solidarity has become a new issue. For example, Senegal decided, in 2007, to eliminate user fees in public healthcare facilities for persons over the age of 60. It could be useful to understand better the social, cultural and religious values underlying the selection of elderly indigents, in order to adapt targeting policies to context.

### A method that does not cover all the indigent

The study revealed a major limitation of the experience: it was not able to ensure acceptable coverage of indigents. The VSCs and, even more, the COGESs were very conservative in their indigent selection processes. However, we do not believe their decisions were biased in favour of important or influential people. Indeed, instructions had been given to ensure a certain neutrality in the VSCs' composition and to avoid having influential leaders (dignitaries, religious leaders, or persons with official status) sitting on them. These recommendations were, in fact, respected [19], although this obviously did not prevent a few rare attempts at influence. Still, these remained the exception and their impact on the committees's choices was marginal. Even though the indigent are predominantly much poorer than the rest of the population, they represent only a very small segment of the population. We expected a larger selection, based on some of the literature on the subject [6,26] as well as on government directives which indicated that the indigent make up between 10% and 20% of the country's population [27]. Also, the United Nations Development Programme considers that indigence corresponds to extreme poverty [28], that is, 9% of the population of Burkina Faso [22]. One might think that, in a context of generalized poverty, this restraint reflects an emic conception of poverty among the populations of that region. However, we tend to believe that the restrictive nature of the selection owes more to the decision to use local and endogenous funding to pay for the exemption. Thus, the limited coverage might reflect not so much the emic perspective of indigence, but rather the COGESs' perception of their limited capacity for endogenous funding. Our study of selection processes showed that some COGESs had influenced the choices of the VSCs because no outside resources had been provided to fund the fees exemption. Other African experiences using a similar strategy reached the same conclusions [29]. One such experience in Mauritania was able to retain only 0.67% of the general population [29], while some in Asia, funded by outside funding agencies, selected more than 20% of the population [30]. In another region of Burkina Faso, the directive given to groups of three people per village to select 20% of the population that they considered the poorest to receive an outside subsidy for the health mutual premium was well respected [11]. Thus, the conflict of interest between identifying the poor and ensuring the financial viability of the services provided is definitely a core factor in explaining the very small coverage of this selection [24].

### Continuation of the experience and the public will

It appears that this community-based process should be retained, because it is efficient and appreciated by the people, but it covers only a very small segment of the population. Thus, the funding approach could be modified in other experiences in order to see how the communities would react to increase the number of people who would be eligible for fees exemption.

To move the COGESs forward in their thinking, one option would be to show them that their cost recovery system generates enough profit to support a certain number of indigents. We have shown that, in the district involved in this study, on average the COGESs had the financial capacity to take on six times more indigents than were retained [19]. This solution would have the advantage of being endogenous and sustainable, but to make it possible, the system's decision-makers would have to demonstrate a much stronger political will. For example, a recent (2007) policy to combat maternal mortality in Burkina Faso instituted a full exemption from delivery user fees, funded by the national budget, for 20% of pregnant women who were considered indigent [27]. Nevertheless, almost none of the health workers are aware of this possibility [31] and the government has done nothing as of yet to determine the best process to select the indigent, even though it had committed to doing so as far back as 1992 [14]. Beginning in 2010, the Ministry of Health has advised the CSPSs to use 200,000 F CFA (considered as a spending ceiling) per year of their own funds to exempt the indigent from user fees [32]. Hopefully, that planning directive will be an incentive to replicate the process we have described in this article.

## Conclusions

Our study showed that the selected indigents lived in the poorest households and were more vulnerable than the rest of the rural population. Still, the basis for eligibility to benefit from fees exemption remains overly restrictive. Further experiences should be considered. Another option, but more long-term, would be to support the creation of a fund to support indigents. This could be based on the standard of 9% of the population. Following the example of Mali and Ghana [9], for example, Burkina Faso has been considering for the past several months the question of whether to implement such arrangements, as it is engaged in planning for national health insurance. However, much research is still needed to define the modalities of such a system, assess its feasibility, and then measure its effectiveness.

## Competing interests

The authors declare that they have no competing interests.

## Authors' contributions

VR, SH, and AB were in charge of the original study design. VR, SH, YK and MO designed the data collection tools. MO, AB and YK were responsible for data collection. BN, MO, SH, and VR conducted the data analysis. All authors contributed to the interpretation of the results. VR and SH wrote the manuscript with contributions from all authors. All authors had full access to all of the data (including statistical reports and tables) in the study and can take responsibility for the integrity of the data and the accuracy of the data analysis.

## Pre-publication history

The pre-publication history for this paper can be accessed here:

http://www.biomedcentral.com/1471-2458/10/631/prepub
